# *Ty*-*6*, a major begomovirus resistance gene on chromosome 10, is effective against Tomato yellow leaf curl virus and Tomato mottle virus

**DOI:** 10.1007/s00122-019-03298-0

**Published:** 2019-02-13

**Authors:** Upinder Gill, John W. Scott, Reza Shekasteband, Eben Ogundiwin, Cees Schuit, David M. Francis, Sung-Chur Sim, Hugh Smith, Samuel F. Hutton

**Affiliations:** 1 Gulf Coast Research and Education Center, Institute of Food and Agricultural Sciences, University of Florida, 14625 CR 672, Wimauma, FL 33598-6101 USA; 2 Nunhems USA, Inc, 890 Embarcadero Drive, West Sacramento, CA 95605 USA; 3 Bejo Zaden, 1749 ZH Warmenhuizen, The Netherlands; 4 Department of Horticulture and Crop Science, The Ohio State University, Ohio Agricultural Research and Development Center, 1680 Madison Ave., Wooster, OH 44691 USA; 5 Department of Bioresources Engineering, Sejong University, 209 Neungdong-ro, Gwangjin-gu, Seoul, 05006 Korea

## Abstract

**Key message:**

*Ty*-*6* is a major resistance gene on chromosome 10 of tomato that provides resistance against monopartite and bipartite begomoviruses and complements resistance conferred by the known *Ty*-*3* and *ty*-*5* genes.

**Abstract:**

Resistance to monopartite and bipartite begomoviruses is an important breeding objective for cultivated tomato. Several begomovirus resistance genes have been introgressed from related *Solanum* species and are available for breeding purposes. In the present study, we mapped an additional locus, *Ty*-*6*, to chromosome 10 of tomato. *Ty*-*6* is effective against both monopartite Tomato yellow leaf curl virus (TYLCV) and bipartite Tomato mottle virus (ToMoV). Gene action is incomplete dominance, with an intermediate resistance response when *Ty*-*6* is heterozygous. Analysis of populations segregating for *Ty*-*6* along with *Ty*-*3* or *ty*-*5* indicates that the highest level of resistance against TYLCV is attained when *Ty*-*6* is combined with an additional resistance allele. Our results also demonstrate that *ty*-*5* is ineffective against ToMoV. Although multiple SNPs linked to *Ty*-*6* were identified and can be used for breeding purposes, none of these were consistently polymorphic between *Ty*-*6* and *ty*-*6* breeding lines. Further research is underway to generate resequencing data for several *Ty*-*6* inbred lines for the discovery of additional sequence polymorphisms that can be used for fine mapping and characterizing the *Ty*-*6* locus.

**Electronic supplementary material:**

The online version of this article (10.1007/s00122-019-03298-0) contains supplementary material, which is available to authorized users.

## Introduction

Begomoviruses, from the family *Geminiviridae*, cause economically significant diseases of major vegetable crops in the world. Two begomoviruses, Tomato yellow leaf curl virus (TYLCV) and African cassava mosaic virus (ACMV), were recently listed among top 10 crop viruses due to their scientific importance and global economic impact (Rybicki 2015). The *Begomovirus* genus is the largest of family *Geminiviridae* and contains more than 200 species (Fauquet et al. 2008). Begomoviruses have either a monopartite or bipartite genome and are transmitted mainly through the insect vector, sweet potato whitefly (*Bemisia tabaci*) (Zhou 2013; Rojas et al. 2005). The begomoviruses that infect cultivated tomato (*Solanum lycopersicum* L.) in the USA and other tropical and subtropical regions include monopartite and bipartite viruses such as TYLCV, Tomato yellow leaf curl Thailand virus (TYLCTHV), Tomato mottle virus (ToMoV), and two newly identified viruses, Tomato leaf deformation virus (ToLDeV) and Tomato leaf curl purple vein virus (ToLCPVV) (Polston and Anderson 1997; Moriones and Navas-Castillo 2000; Macedo et al. 2018; Melgarejo et al. 2013). Management strategies for begomoviruses rely heavily on insecticide treatments to control whiteflies, but such measures can be ineffective because of the development of resistance against insecticides in the vector (Moriones and Navas-Castillo 2000; Omer et al. 1993).

Use of genetic resistance against begomoviruses has proved successful in tomato. Tomato breeding strategies have primarily focused on the introgression of resistance alleles from related wild germplasm. Resistance has been identified in a number of *Solanum* species, including *S. pimpinellifolium*, *S. peruvianum*, *S. chilense*, *S. habrochaites* and *S. cheesmaniae* (Ji et al. 2007; Picó et al. 1996; Scott 2006), and several resistance genes have been introgressed and genetically characterized in tomato. *Ty*-*1* was introgressed from *S. chilense* accession LA1969 and mapped to chromosome 6 of tomato (Zamir et al. 1994; Verlaan et al. 2011). Another TYLCV resistance gene, *Ty*-*3*, was introgressed from *S. chilense* accessions, LA1932/LA2779/LA1938, and also mapped to chromosome 6 (Ji et al. 2007). Later, *Ty*-*1* and *Ty*-*3* were determined to be allelic and to code for an RNA-dependent RNA polymerase, which imparts resistance by increasing cytosine methylation of viral genomes (Verlaan et al. 2013; Butterbach et al. 2014). The second TYLCV resistance gene discovered was *Ty*-*2*, which was introgressed on chromosome 11 from *S. habrochaites* (Hanson et al. 2006; Yang et al. 2014; Yamaguchi et al. 2018). Yamaguchi et al. (2018) recently determined that *Ty*-*2* is a nucleotide-binding domain and leucine-rich repeat-containing (NB-LRR) gene (Yamaguchi et al. 2018). In addition to *Ty*-*1* and *Ty*-*3*, tomato lines derived from *S. chilense* accessions exhibited multi-genic control of TYLCV resistance, indicating the presence of additional resistance loci in studied introgressed lines (Scott et al. 1996). Later, another TYLCV resistance gene, *Ty*-*4,* was identified from *S. chilense* accession, LA1932, and mapped on chromosome 3 (Ji et al. 2009). Compared to other *Ty* genes, *Ty*-*4* is less effective against TYLCV (Kadirvel et al. 2013). A recessive resistance to TYLCV derived from the cultivar ‘Tyking’ was also observed in some of the tomato breeding lines (Hutton et al. 2012). This recessive resistance was mapped on chromosome 4 as *ty*-*5* locus (Hutton et al. 2012; Anbinder et al. 2009). *ty*-*5* encodes a messenger RNA surveillance factor Pelota (Pelo) which is involved in ribosome recycling phase of protein synthesis (Lapidot et al. 2015).

Besides the above-mentioned genes, recent studies provide evidence for the presence of another, unidentified, resistance gene(s). For example, Hutton et al. (2012) found that TYLCV resistance in the *ty*-*5* parental lines, Fla. 8753 and Fla. 344, was significantly greater than that of F_2_ progeny which were homozygous for *ty*-*5*, and F_3_ progeny lines derived from Fla. 8753 also demonstrated segregation for resistance that was not due to *ty*-*5* (Hutton et al. 2012). Verlaan et al. (2013) likewise indicated the presence of additional resistance allele in Fla. 8680 (Verlaan et al. 2013). It is likely that the putative resistance allele reported by Hutton et al. (Hutton et al. 2012) in Fla. 8383 and in Fla. 8638B is the same as that in Fla. 8680, since Fla. 8680 is the resistant parent in a cross from which Fla. 8383 is derived (S. Hutton, Unpublished). This resistance allele is presumably inherited from *S. chilense* and was recently designated as *Ty*-*6* (Scott et al. 2015). Here, we report the genetic mapping of *Ty*-*6*, and we characterize the effect of *Ty*-*6* alone and in combination with other resistance genes.

## Materials and methods

### Plant materials and experimental design

Six large-fruited, fresh market tomato breeding lines with moderate or high levels of begomovirus resistance were used as donor parents to develop F_2_ populations segregating for resistance. Breeding lines with resistance derived from *S. chilense* accession LA2779 (LA2779) included Fla. 8680, Fla. 8383 and Fla. 8503C. Fla. 8680 expresses a high level of resistance that is based on *Ty*-*3* and on another locus (loci) (Verlaan et al. 2013). Fla. 8383 and Fla. 8503C were each selected from a cross between Fla. 8680 and a susceptible breeding line; each expresses a moderate level of resistance that is not based on *Ty*-*3*. Fla. 8472B, Fla. 8638B and Fla. 8382B have high levels of resistance derived from *S. chilense* accession LA1938 (LA1938) and from ‘Tyking’; Fla. 8638B and Fla. 8382B are full-sib lines, and resistance in both lines is conferred, in part, by *ty*-*5* from ‘Tyking’ (Hutton et al. 2012). Breeding lines Fla. 7776, Fla. 7060, Fla. 7987, Fla. 8059, Fla. 8044 and Fla. 7781 were used as susceptible parents for the development of F_2_ populations for mapping and characterization of the *Ty*-*6* locus. Each of these lines is further described in Supplementary Table S1.

Fla. 8383 was crossed to Fla. 7776, and an F_2_ population was developed for the initial genetic mapping of *Ty*-*6*; subsequently, Fla. 8503C was crossed to Fla. 7060 to develop a population for *Ty*-*6* location confirmation and for genetic mapping of ToMoV resistance. Fla. 8382B was crossed to Fla. 8059 and Fla. 8638B was crossed to Fla. 7987 to study the effect of *Ty*-*6* in combination with *ty*-*5* on TYLCV resistance; Fla. 8472B was crossed to Fla. 8044 to study the effect of *Ty*-*6* in combination with *ty*-*5* on ToMoV resistance. Fla. 8680 was crossed to Fla. 7781 to study the effect of *Ty*-*6* in combination with *Ty*-*3* on TYLCV resistance. All experiments were conducted in the field using randomized complete block design with three blocks and with six plant plots for each parent and ~ 40 plant plots for F_2_ populations.

For haplotyping, in addition to above listed lines, additional resistant and susceptible lines were included. Resistant lines Fla. 8753, Fla. 344, Fla. 8624 and Fla. 8062 were derived from ‘Tyking’ and LA1938 (Hutton et al. 2012; Scott et al. 2015). Fla. 8753, Fla. 344 and Fla. 8062 each have a high level of resistance due to the presence of *ty*-*5* and *Ty*-*6*. Fla. 8624 contains only *Ty*-*6* and has a moderate level of resistance to TYLCV. Fla. 7804 and Fla. 8022 are large-fruited susceptible inbreds.

### Inoculation and disease evaluation

All inoculations were performed using whiteflies viruliferous for TYLCV or ToMoV according to the method developed by Griffiths and Scott (Griffiths and Scott 2001) with some modifications. Briefly, seedlings 4 weeks past the cotyledon stage (three to four leaves) were exposed to viruliferous whiteflies for 2 weeks in growth chambers. For these purposes, separate viruliferous whitefly colonies were maintained in temperature-controlled growth rooms on TYLCV- or ToMoV-infected tomato plants, and whitefly-infested plants were transferred to separate growth rooms for each inoculation. Growth rooms were maintained at 25 °C with a 14 h photoperiod. On average, 20 whiteflies per plant were used during inoculation of tomato breeding lines and F_2_ populations. Following inoculation, the whiteflies were killed by treating plants with an insecticidal soap and with imidacloprid [Admire (Bayer CropScience, Research Triangle Park, NC)], prior to transplanting to the field. Plants were rated for disease severity in the field approximately 40 days after exposure to whiteflies on a 0–4 disease severity index (DSI) scale as described previously (Scott et al. 1996), where 0 = no symptoms and 4 = severe symptoms and stunting. Intermediate scores such as 1.5 and 2.5 were incorporated to allow for more precise disease severity ratings.

### DNA extraction and molecular marker genotyping

DNA was extracted from young leaves of individual plants using a modified cetyltrimethylammonium bromide (CTAB) procedure (Fulton et al. 1995). Leaf samples for DNA isolation were collected from plants at seedling stage before transplanting to field.

For genotyping, single-nucleotide polymorphism (SNP) information generated by the Solanaceae Coordinated Agricultural Project (SolCAP) was used to identify a subset of 384 SNP markers optimized for genotyping fresh market tomatoes (Hamilton et al. 2012; Sim et al. 2012a, b). SNP markers were optimized by filtering data generated from the SolCAP Infinium Array (Illumina, Foster City, CA) based on allele frequency for 140 tomatoes annotated as contemporary fresh market varieties (Sim et al. 2012b). Markers with high polymorphic information content (PIC) were retained. Genetic positions based on Sim et al. (2012a) were used to select SNPs distributed across all 12 chromosomes. Finally, gaps in genome coverage were filled in by selecting high PIC markers based on physical position. For the initial mapping, the 384-SNP panel was used to genotype 203 TYLCV-inoculated F_2_ plants and their parents, Fla. 7776 and Fla. 8383, using competitive allele-specific PCR genotyping chemistry (Supplementary Table S2; KASP, www.lgcgroup.com).

For confirmation of TYLCV resistance QTL detected in the Fla. 7776 × Fla. 8383 population and for mapping ToMoV resistance, an independent F_2_ population from the cross between Fla. 7060 and Fla. 8503C was genotyped with chromosome 10 specific markers. Prior to inoculation, transplants of this population were separated into two groups: one for phenotyping with TYLCV and the other for phenotyping with ToMoV. Both groups were genotyped in cooperation with Nunhems USA, Inc. (www.nunhemsusa.com) and Bejo Seeds, Inc. (www.bejoseeds.com) using proprietary markers corresponding to the *Ty*-*6* interval on chromosome 10 (Supplementary Table S3). To study the effect of combinations of different *Ty* genes on TYLCV or ToMoV resistance in other listed populations, individual plants were genotyped with one or more of the following: the proprietary marker, B_04 for *Ty*-*6*; UF_10.61192 for *Ty*-*6* which corresponds to the solcap_snp_sl_61192 (Sim et al. 2012a); *Sl*NAC1 (Anbinder et al. 2009) and TY5.2 markers for *ty*-*5*; and TY3-5 for *Ty*-*3* (Supplementary Table S4).

### Genetic mapping, QTL analysis and statistical analysis

Genotyping data of 156 SNP markers tested on the (Fla. 8383 × Fla. 7776) F_2_ population were used for linkage map construction using JoinMap 4.1 (Van Ooijen 2006). A threshold recombination frequency of < 0.25 was used for grouping loci into linkage groups. For QTL detection, linkage map information generated by JoinMap 4.1 was used for analysis in QTL Cartographer version 2.5 (Wang et al. 2012). QTLs were identified by single-locus QTL analysis using interval mapping. The threshold LOD scores were calculated using 1000 permutations as given in the software (Churchill and Doerge 1994). A minimum LOD score of > 3.0 was used to declare a QTL.

Disease severity data were analyzed using a Wald-type statistics (WTS) procedure for nonparametric ordinal data (Shah and Madden 2004). The overall effect of *Ty*-*6* alone and in combination with *Ty*-*3* or *ty*-*5* on TYLCV and/or ToMoV resistance was calculated by WTS and analysis of variance type statistics (ATS) on ranked data using PROC MIXED procedure in SAS (version 9.4; SAS Institute, Cary, NC, USA). Relative marginal effects (RME) and 95% confidence intervals were calculated according to the procedure given previously (Shah and Madden 2004).

## Results

### The TYLCV resistance locus, *Ty*-*6*, maps to chromosome 10 of tomato

**Fig. 1:**
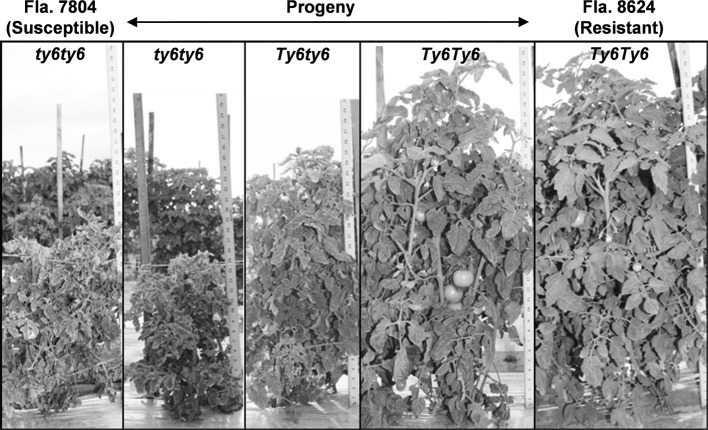
TYLCV disease severity on the susceptible parent, Fla. 7804; the *Ty*-*6* resistant parent, Fla. 8624 (containing *Ty*-*6*); and F_2_ plants with different *Ty*-*6* genotypes

**Fig. 2:**
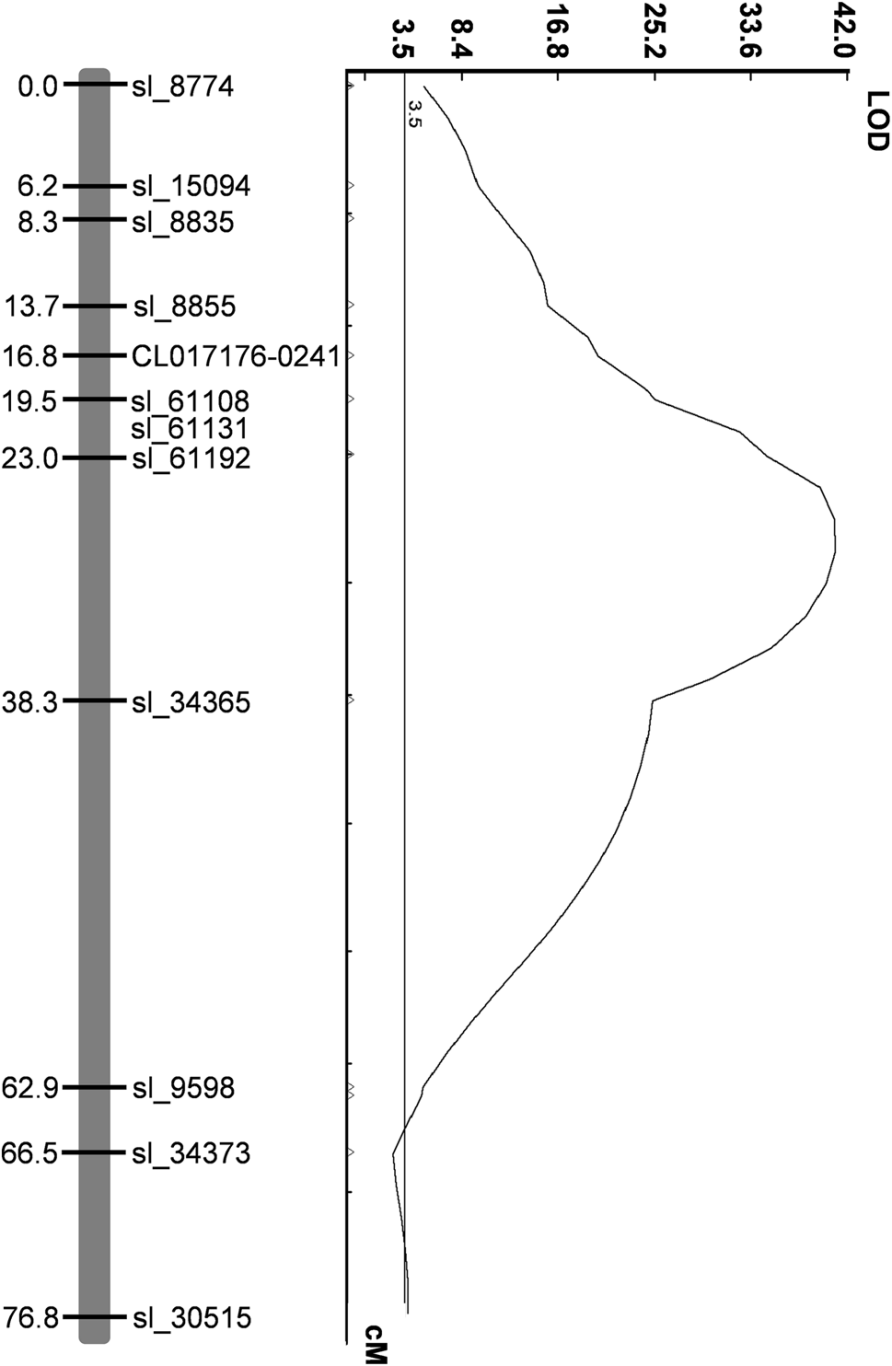
LOD plot from simple interval mapping analysis of TYLCV disease severity on F_2_ population from the cross between Fla. 8383 and Fla. 7776 and genotyped with 158 polymorphic SolCAP SNP markers indicated the presence of a single QTL on chromosome 10 of tomato at LOD score of 41.1

Fla. 8383 is a fresh market breeding line with moderate resistance to TYLCV derived from *S. chilense* accession LA2779. As this line does not contain any previously known TYLCV resistance genes, we crossed Fla. 8383 with the susceptible parent, Fla. 7776, to develop an F_2_ mapping population. Resistance in the F_1_ was intermediate to the parents, indicating incomplete dominance (Table 1). Additionally, segregation among F_2_ individuals generally followed three phenotypic levels, similar to that observed for the parents and the F_1_ (Fig. 1). The F_2_ population of 203 individuals was genotyped using a 384 SNP array, of which 158 SNPs were confirmed polymorphic between the parents and segregating in the population. Linkage analysis generated a total map distance of 878.4 cM for all chromosomes except chromosome 11 (which had no polymorphic SNPs). Interval mapping identified *Ty*-*6* as a single QTL on chromosome 10 explaining 59.4% of the phenotypic variance with LOD score of 41.1 (Fig. 2). The SNP marker solcap_snp_sl_61192 showed the strongest association with the phenotype. No evidence of any other QTL associated with TYLCV resistance was found on other chromosomes (Supplementary Fig. S1).

**Table 1:** Mean disease severity index (DSI) of TYLCV-resistant parent, Fla. 8383, TYLCV-susceptible parent, Fla. 7776, and F_1_ plants

Population	*Ty*-*6*	*N*	Mean DSI^a^	RME	95% CI
Fla. 7776	–	12	3.9a	0.81944	0.74263–0.83142
F_1_ (Fla. 8383 × Fla 7776)	+−	12	3.0b	0.51389	0.48664–0.54096
Fla. 8383	++	12	2.0c	0.16667	0

^a^DSI based on a 0–4 scale where higher numbers indicate more severe virus symptoms; mean separations within either viral trial are based on 95% confidence intervals (CI) of relative marginal effects (RME) on ranked DSI data

### *Ty*-*6* confers resistance against ToMoV

**Fig. 3:**
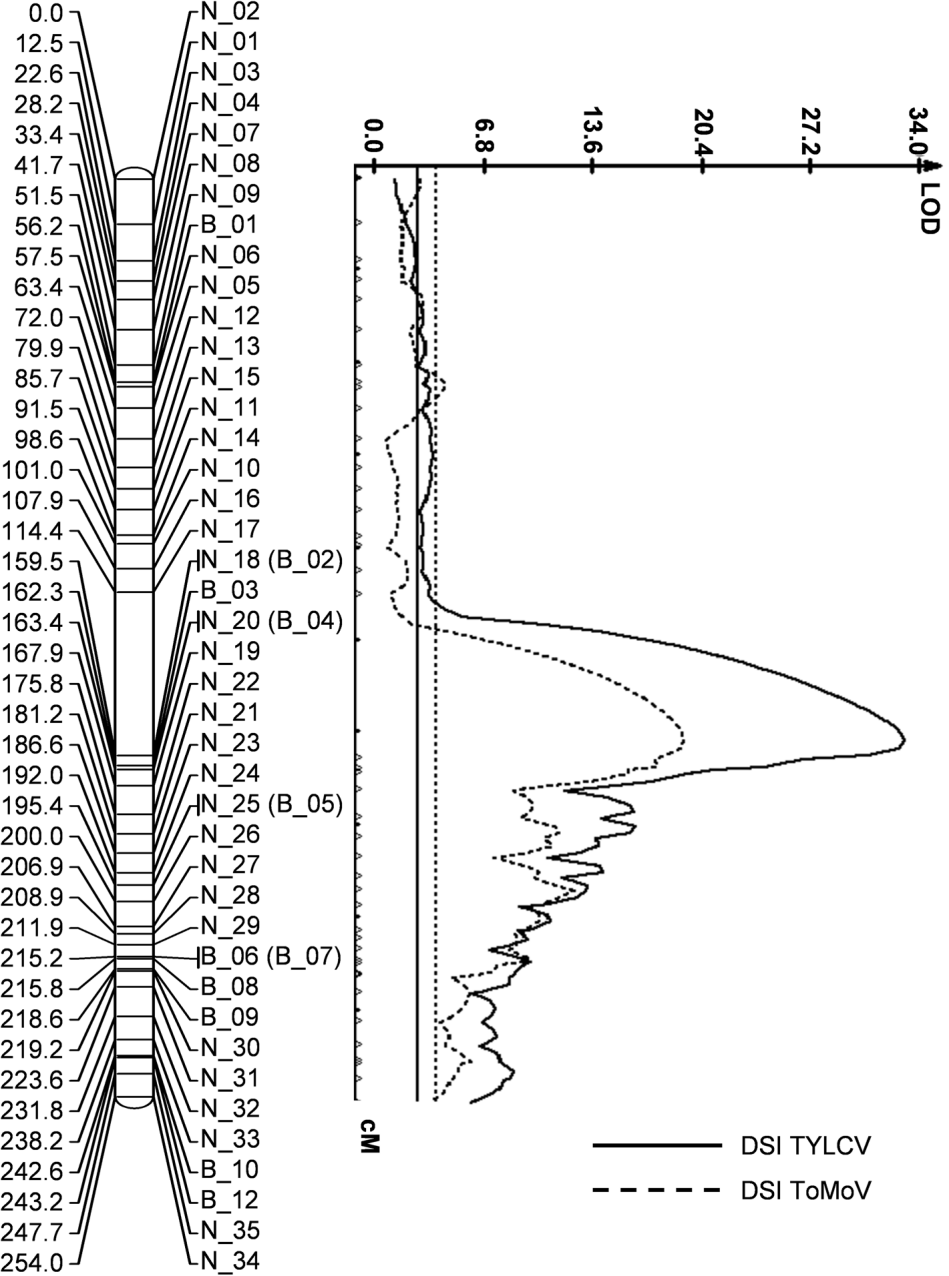
LOD plots from simple interval mapping analysis of TYLCV and ToMoV disease severity on independent subsets of the F_2_ population from the cross between Fla. 7060 and Fla. 8503C and genotyped with chromosome 10 specific proprietary SNP markers. The QTLs for TYLCV and ToMoV resistance were identified on chromosome 10 with LOD scores of 33.19 and 19.36, respectively

In addition to TYLCV, breeding lines carrying *Ty*-*6* are also resistant to ToMoV. To further confirm the *Ty*-*6* locus for resistance to TYLCV and to test for an effect of this gene against a bipartite begomovirus, an F_2_ population developed from the cross between Fla. 7060 and Fla. 8503C was used. F_2_ plants were divided into two groups: one (110 F_2_ plants) of which was inoculated with TYLCV and the other (114 F_2_ plants) inoculated with ToMoV. Both sets of F_2_ plants were genotyped with same set of chromosome 10 specific molecular markers. QTL analyses identified the same locus on chromosome 10 in both groups of F_2_ plants with LOD scores of 33.19 for TYLCV and 19.36 for ToMoV, demonstrating the efficacy of *Ty*-*6* against both TYLCV and ToMoV (Fig. 3). The markers N_18 (and B_02) explained 97.57% of phenotypic variance for TYLCV and 99.44% of phenotypic variance for ToMoV. Resistance conferred by *Ty*-*6* against TYLCV and ToMoV was additive in nature, confirming incompletely dominant inheritance (Tables 2, 3).

**Table 2:** Statistical analysis of F_2_ population from the cross between Fla. 7060 and Fla. 8503C for an effect of the *Ty*-*6* locus in tomato on TYLCV and ToMoV disease severity

Population	Source	Num DF	Den DF	*χ* ^2^	*F* value	Pr > *χ*^2^
7060 × 8503C (TYLCV)	*Ty*-*6*	2	107	204.04	102.02	< 0.0001***
7060 × 8503C (ToMoV)	*Ty*-*6*	2	111	94.36	47.18	< 0.0001***

**Table 3:** Mean disease severity index (DSI) and relative marginal effect (RME) for TYLCV and ToMoV disease severity on *Ty*-*6* genotypes in F_2_ populations from the cross between Fla. 7060 and Fla. 8503C

Population	*Ty*-*6*^a^	*N*	Mean DSI^b^	RME	95% CI
7060 × 8503C (TYLCV)	–	33	3.8a	0.75496	0.69438–0.79437
	+−	57	3.2b	0.47727	0.44655–0.50876
	++	20	2.3c	0.14409	0.11554–0.20116
7060 × 8503C (ToMoV)	–	30	3.2a	0.72836	0.66903–0.77317
	+−	54	2.8b	0.52615	0.48853–0.56273
	++	30	2.2c	0.22456	0.18101–0.29724

^a^*Ty*-*6* genotype based on proprietary marker B_04

^b^DSI based on a 0–4 scale where higher numbers indicate more severe virus symptoms; mean separations within either viral trial are based on 95% confidence intervals (CI) of relative marginal effects (RME) on ranked DSI data. TYLCV DSI for Fla. 7060 = 3.8, Fla. 8503C = 2.5; ToMoV DSI for Fla. 7060 = 3.4, Fla. 8503C = 2.2

### Effect of *Ty*-*6* in combination with *Ty*-*3* or *ty*-*5*

In order to understand interactions among *Ty* resistance genes, we studied F_2_ breeding populations segregating for *Ty*-*6* with *Ty*-*3* or *ty*-*5*, the latter of which are both effective against TYLCV (Verlaan et al. 2013; Lapidot et al. 2015). The Fla. 8680 × Fla. 7781 population was genotyped for *Ty*-*3* and *Ty*-*6* and phenotyped for TYLCV. Results indicated significant associations of TYLCV resistance with both *Ty*-*6* (*P* < 0.0001) and *Ty*-*3* genes (*P* < 0.0001) (Table 4). The interactions between *Ty*-*6* and *Ty*-*3* were nonsignificant indicating no involvement of epistatic effects. Segregation in the population indicated complementary action of *Ty*-*3* and *Ty*-*6* for resistance to TYLCV (Table 5). Although both genes effectively reduced TYLCV disease severity, *Ty*-*3* provided a stronger resistance than *Ty*-*6* (Table 5). Homozygosity for *Ty*-*3* produced a comparable level of resistance to heterozygosity for both *Ty*-*3* and *Ty*-*6*, but otherwise, greater levels of resistance were achieved with two-gene combinations than with either gene alone. The highest level of resistance was observed in plants homozygous for both *Ty*-*3* and *Ty*-*6*.

**Table 4:** Statistical analysis of F_2_ population developed from the cross between Fla. 8680 and Fla. 7781 for effect of the *Ty*-*3* and the *Ty*-*6* loci in tomato on disease severity of TYLCV

Population	Source	Num DF	Den DF	*χ* ^2^	*F* value	*Pr* > *χ*^2^
8680 × 7781	*Ty*-*3*	2	650	441.71	220.86	< 0.0001***
	*Ty*-*6*	2	650	107.55	53.78	< 0.0001***
	*Ty*-*3 *×* Ty*-*6*	4	650	4.46	1.12	0.35

**Table 5:** Mean disease severity index (DSI) and relative marginal effect (RME) for TYLCV disease severity on genotypes segregating for the *Ty*-*3* and the *Ty*-*6* loci in F_2_ population from the cross between Fla. 8680 and Fla. 7781

Population	*Ty*-*3*^a^	*Ty*-*6*^a^	*N*	Mean DSI^b^	RME	95% CI
8680 × 7781	–	–	52	3.6a	0.87992	0.85381–0.90011
	–	+−	92	3.3b	0.79552	0.76176–0.82356
	–	++	31	2.7c	0.62343	0.53244–0.70569
	+−	–	81	2.5c	0.57059	0.52830–0.61157
	+−	+−	177	2.1d	0.44419	0.41850–0.47046
	+−	++	75	1.9e	0.32278	0.28646–0.36218
	++	–	40	1.9d	0.40641	0.34100–0.47584
	++	+−	74	1.5f	0.25861	0.22145–0.30130
	++	++	37	1.3g	0.18345	0.14940–0.22477

^a^*Ty*-3 genotype based on marker TY3-5 and *Ty*-*6* genotype based on marker UF_10.61192

^b^DSI based on a 0–4 scale where higher numbers indicate more severe virus symptoms; mean separations within either viral trial are based on 95% confidence intervals (CI) of relative marginal effects (RME) on ranked DSI data

Two F_2_ populations derived from crosses Fla. 8059 × Fla. 8382B and Fla. 7987 × Fla. 8638B were analyzed to characterize the resistance response of *Ty*-*6* in combination with *ty*-*5* against TYLCV. In both populations, resistance was significantly associated with *Ty*-*6* (*P* < 0.0001) and *ty*-*5* (*P* < 0.0001); interactions were also significant between *Ty*-*6* and *ty*-*5* in the tested populations probably due to the recessive nature of *ty*-*5* (Table 6). As with the *Ty*-*3* and *Ty*-*6* combination, *Ty*-*6* and *ty*-*5* likewise provided complementary resistance, and results were similar for both populations (Table 7). Plants homozygous for *Ty*-*6* were equally resistant as plants homozygous for *ty*-*5*, and heterozygosity at the *ty*-*5* locus provided no control, which is consistent with it being a recessive gene. Again, the highest level of disease control was observed in plants with two-gene combinations, and plants homozygous for both genes were more resistant than those homozygous for *ty*-*5* and heterozygous for *Ty*-*6*.

**Table 6:** Statistical analysis of F_2_ populations developed from the crosses between Fla. 8059 and Fla. 8382B, and between Fla. 7987 and Fla. 8638B for effect of the *ty*-*5* and the *Ty*-*6* loci in tomato on disease severity of TYLCV

Population	Source	Num DF	Den DF	*χ* ^2^	*F* value	*Pr* > *χ*^2^
8059 × 8382B	*ty*-*5*	2	183	170.64	85.32	< 0.0001***
	*Ty*-*6*	2	183	112.99	56.50	< 0.0001***
	*ty*-*5 *×* Ty*-*6*	4	183	10.63	2.66	0.0310*
7987 × 8638B	*ty*-*5*	2	208	276.01	138.01	< 0.0001***
	*Ty*-*6*	2	208	204.72	102.36	< 0.0001***
	*ty*-*5 *×* Ty*-*6*	4	208	12.83	0.014	0.0139*

**Table 7:** Mean disease severity index (DSI) and relative marginal effect (RME) for TYLCV disease severity on genotypes segregating for the *ty*-*5* and the *Ty*-*6* loci in F_2_ populations from the crosses between Fla. 8059 and Fla. 8382B, and between Fla. 7987 and Fla. 8638B

Population	*ty*-*5*^a^	*Ty*-*6*^a^	*N*	Mean DSI^b^	RME	95% CI
8059 × 8382B	–	–	16	3.6a	0.85270	0.74898–0.90865
	–	+−	14	2.9b	0.59487	0.48097–0.69795
	–	++	12	2.1c	0.34549	0.26532–0.43726
	+−	–	25	3.4a	0.76563	0.68183–0.82655
	+−	+−	47	2.9b	0.61209	0.56035–0.65959
	+−	++	33	2.2c	0.39915	0.34840–0.45333
	++	–	10	2.1c	0.36823	0.28538–0.46083
	++	+−	26	1.4d	0.15805	0.12912–0.19839
	++	++	9	1.0d	0.11227	0.05253–0.26289
7987 × 8638B	–	–	21	3.7a	0.82960	0.75382–0.87932
	–	+−	33	2.9b	0.62861	0.58829–0.66653
	–	++	17	2.1c	0.37829	0.31494–0.44699
	+−	–	28	3.7a	0.84126	0.79790–0.87214
	+−	+−	27	2.8b	0.59908	0.53552–0.65852
	+−	++	19	2.3c	0.40893	0.35758–0.46279
	++	–	22	2.1c	0.36741	0.28618–0.45939
	++	+−	30	1.4d	0.20914	0.16873–0.26180
	++	++	20	0.9e	0.10230	0.07905–0.14019

^a^*ty*-*5* and *Ty*-*6* genotypes are based on markers, TY5.2 and proprietary marker B_04, respectively, in cross 8059 × 8382B. In cross 7987 × 8638B, *ty*-*5* and *Ty*-*6* genotypes are based on marker *Sl*NAC1 and UF_10.61192, respectively

^b^DSI based on a 0–4 scale where higher numbers indicate more severe virus symptoms; mean separations within either viral trial are based on 95% confidence intervals (CI) of relative marginal effects (RME) on ranked DSI data. TYLCV DSI for Fla. 8059 = 4.0, Fla. 8382B = 0.5, Fla. 7987 = 4.0, Fla. 8638B = 0.5

The effect of *Ty*-*6* in combination with *ty*-*5* was also evaluated against ToMoV using an F_2_ population from the cross Fla. 8044 × Fla. 8472. Results demonstrated that *Ty*-*6* has a significant effect toward reducing ToMoV disease severity (Table 3). However, neither *ty*-*5* nor its interaction with *Ty*-*6* had any significant effect on TYLCV disease severity, demonstrating that *ty*-*5* is ineffective against ToMoV (Table 8). Similar to the population from a cross Fla. 7060 × Fla. 8503C, the current population also indicated incomplete dominant inheritance of *Ty*-*6* in resistance against ToMoV (Table 9).

**Table 8:** Statistical analysis for effects of *ty*-*5* and *Ty*-*6* toward ToMoV resistance in F_2_ population developed from the cross between Fla. 8044 and Fla. 8472

Population	Source	Num DF	Den DF	*χ* ^2^	*F* value	*Pr* > *χ*^2^
8044 × 8472	*ty*-*5*	2	134	0.19	0.10	0.9081
	*Ty*-*6*	2	134	15.52	7.76	0.0004***
	*ty*-*5 *×* Ty*-*6*	4	134	3.75	0.94	0.4404

**Table 9:** Mean disease severity index (DSI) and relative marginal effect (RME) for ToMoV disease severity on genotypes segregating for the *ty*-*5* and the *Ty*-*6* loci in F_2_ population from the cross between Fla. 8044 and Fla. 8472

Population	*ty*-*5*^a^	*Ty*-*6*^a^	*N*	Mean DSI^b^	RME	95% CI
8044 × 8472	–	–	11	2.6a	0.63724	0.40941–0.81161
	–	+−	15	1.9ab	0.50452	0.38414–0.62424
	–	++	10	1.4b	0.39371	0.26406–0.54350
	+−	–	22	2.2a	0.56294	0.45312–0.66472
	+−	+−	32	1.7ab	0.51814	0.43916–0.59564
	+−	++	18	1.4ab	0.42347	0.32013–0.53645
	++	–	8	2.7a	0.71933	0.48409–0.86402
	++	+−	17	1.4ab	0.43007	0.29219–0.58305
	++	++	10	0.7b	0.29371	0.17496–0.45910

^a^*ty*-5 genotype based on marker TY5.2 and *Ty*-*6* genotype based on marker B_16

^b^DSI based on a 0–4 scale where higher numbers indicate more severe virus symptoms; mean separations within either viral trial are based on 95% confidence intervals (CI) of relative marginal effects (RME) on ranked DSI data. ToMoV DSI for Fla. 8044 = 3.2, Fla. 8472 = 0.0

### Haplotype differences among TYLCV-resistant and TYLCV-susceptible tomato germplasm

**Fig. 4:**
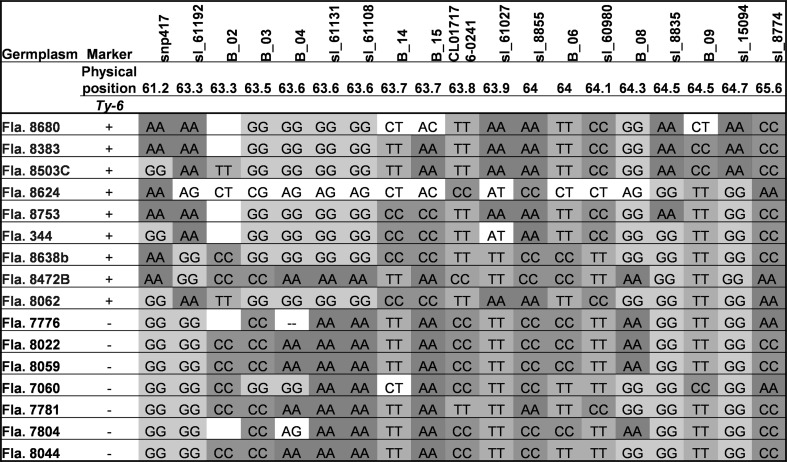
*Ty*-*6* marker haplotyping of select University of Florida breeding lines exhibiting resistance (green text) and susceptibility (red text) to TYLCV. All breeding lines were tested for the presence and absence of *Ty*-*6*. The physical location (in Mega bases) of molecular markers is based on version SL3.0 of the tomato genome. (Abbreviations: sl = solcap_snp_sl; B = Bejo proprietary marker; snp417 = SGN-U317657_C2_At3g47930_snp417)

Tomato breeding programs regularly rely on marker-assisted selections in order to identify resistant plants without conducting virus inoculations or performing evaluations under conditions of high disease pressure. With the goal of identifying molecular markers that consistently distinguish between *Ty*-*6* and susceptible genotypes, nine resistant and seven susceptible breeding lines were surveyed genotypically with 19 SNP markers corresponding to the *Ty*-*6* physical region (Fig. 4). None of the SNP markers, however, consistently distinguished between resistant and susceptible genotypes. Although the SNP markers solcap_snp_sl_61131 and solcap_snp_sl_61108 correctly identified the *Ty-6* genotype among most of the breeding lines surveyed, these markers did not detect the resistant allele in Fla. 8472B, and they also failed to consistently distinguish between all susceptible and resistant lines in a broader panel of germplasm that was tested (data not shown). Thus, although these markers are useful within specific populations, they may have limited utility for consistently tracking *Ty*-*6* across diverse germplasm.

## Discussion

Disease resistance against begomoviruses is one the most important breeding objectives in tomato breeding programs across the world. The University of Florida, Institute of Food and Agricultural Sciences (UF/IFAS) tomato breeding program initiated its begomovirus resistance project in the early 1990s, after ToMoV emerged and caused significant crop losses in the state (Scott et al. 1996; Scott and Schuster 1991). Early disease screens identified several ToMoV resistant *S. chilense* accessions (i.e., LA1932, LA1938, LA1961, LA1968, and LA2779), which were used for introgression breeding (Scott and Schuster 1991; Scott et al. 1996). Throughout subsequent selection cycles, these breeding materials were screened with ToMoV, and once TYLCV was discovered in Florida, lines were screened separately with the two viruses. These efforts led to the genetic mapping and cloning of *Ty*-*3*, a major TYLCV resistance gene on chromosome 6 derived independently from two *S. chilense* accessions, LA1932 and LA2779 (Ji et al. 2007; Verlaan et al. 2013). *Ty*-*3* was thought to contribute partial resistance to ToMoV, and a minor role for this gene against ToMoV has been confirmed (Ji et al. 2007). Further analysis of breeding lines with resistance derived from LA1932 led to the identification of the *Ty*-*4* resistance locus on chromosome 3 (Ji et al. 2009), and screening of breeding material carrying *Ty*-*3* alone or in combination with *Ty*-*4* against multiple bipartite begomoviruses indicated greater resistance with the two-gene combination (Nakhla et al. 2004). However, it was not clear if other bipartite resistance loci besides *Ty*-*4* may be present in advanced breeding materials.

More recently, several studies have provided supporting evidence for an additional locus from *S. chilense*. For example, phenotypic analysis of selected *Ty*-*3* recombinant inbred lines derived from a cross with Fla. 8680 indicated partial TYLCV resistance in several RILs that lacked *Ty*-*3* (Verlaan et al. 2013). Variation for resistance that was not due to *ty*-*5* or to any other known locus was also described in Fla. 8753 and Fla. 344, both of which derive their resistance from the hybrid, ‘Tyking’ and from *S. chilense* accession LA1938 (Hutton et al. 2012). Additionally, Hutton et al. (2012) described genetic resistance in the inbred lines Fla. 8638B and Fla. 8383 that was likewise not due to any known resistance locus (Hutton et al. 2012). Scott et al. (2015) recently designated the new resistance locus in Fla. 8638B, Fla. 8624 and Fla. 8680 as *Ty*-*6*, and efforts have been ongoing to map this locus in tomato (Scott et al. 2015).

In the present study, an F_2_ population derived from Fla. 8383 and inoculated with TYLCV was used to map *Ty*-*6* to the distal end of chromosome 10. This locus was then confirmed in multiple populations for its effect against TYLCV as well as ToMoV. Although the exclusive use of whitefly-mediated inoculations in the present study could suggest an effect of *Ty*-*6* against the insect vector, Caro et al. (2015) showed that Fla. 8383 is also resistant against Agrobacterium-mediated TYLCV inoculation, demonstrating that the resistance conferred by *Ty*-*6* is not against the vector but against begomoviruses. Using a RIL population developed from the cross between Fla. 456 and a susceptible line, CLN1621L, Kadirvel et al. (2013) identified four QTLs on chromosomes 4, 6, 10 and 11 for resistance to Tomato yellow leaf curl Thailand virus Taiwan strain (TYLCTHV-TW). Of these, the QTLs on chromosomes 4 and 10 explained the greatest amount of phenotypic variation. Whereas the chromosome 4 QTL likely corresponded to the *ty*-*5* locus, the other QTL mapped between 61 and 63 Mb on chromosome 10 (Kadirvel et al. 2013). Several markers corresponding to the same interval and used in the present study (e.g., B_02 and N_18) demonstrated very strong linkage with *Ty*-*6*. Fla. 456 is a UF/IFAS breeding line with resistance derived from *S. chilense* accession LA2779 and from ‘Tyking’ (Bian et al. 2007). Based on this pedigree and the current genetic mapping information, it is likely that the QTL on chromosome 10 in Fla. 456 is due to the presence of *Ty*-*6*. This line has consistently displayed high levels of resistance against TYLCTHV, Tomato leaf curl Taiwan virus (ToLCTWV) and other predominant begomoviruses in Taiwan, Senegal, Mali, south India, Indonesia, the Philippines and El Salvador (Kadirvel et al. 2013; Chomdej et al. 2007). It is likely that *Ty*-*6* is involved in each of these resistance responses, but further research is needed to understand whether these responses are due to *ty*-*5*, to *Ty*-*6* or to the combination of the two genes.

The identification of *Ty*-*6* is an important finding, not only because it expands the toolkit of *Ty* genes available to breeders, but also because it confers resistance to both monopartite and bipartite begomoviruses. Our results clearly demonstrate that *Ty*-*6* is effective against both TYLCV and ToMoV. Although *Ty*-*3* was once considered to be highly effective against ToMoV, our findings, along with those presented by Scott et al. (2015), suggest that ToMoV resistance in lines such as Fla. 8680 is due primarily to the contribution of *Ty*-*6* (Agrama and Scott 2006; Ji et al. 2007; Scott et al. 2015). Likewise, although *ty*-*5* and *Ty*-*6* collectively contribute to TYLCV in lines such as Fla. 8638B and Fla. 8472, we found that *ty*-*5* is completely ineffective against ToMoV, and the bipartite resistance in such lines is due rather to the presence of *Ty*-*6* (Table 8). This, however, does not imply that *ty*-*5* is ineffective against all bipartite begomoviruses. It is very likely that the recessive resistance locus, *tcm*-*1*, derived from ‘Tyking’ is the same as *ty*-*5*, and this locus conferred resistance to the bipartite *Tomato chlorotic mottle virus* (Giordano et al. 2005). Similarly, the chromosome 4 QTL identified by Kadirvel et al. (2013) conferred resistance to the bipartite TYLCTHV-TW. Thus, although *ty*-*5* is useful against TYLCV and many other viruses, it does not have efficacy against all bipartite begomoviruses.

Scott et al. (2015) reported *S. chilense* as the source of *Ty*-*6* in Fla. 8624 and Fla. 8638B, and this is likely true, considering that this species is in the pedigree of all *Ty*-*6*-containing UF/IFAS lines tested so far. Our results cannot verify this, however, since none of the markers tested consistently distinguishes resistant and susceptible haplotypes, and there is no evidence supporting the presence of a large introgression. Wild species introgressions are often accompanied by linkage with genes that negatively affect horticultural performance (termed, linkage drag). Such was the case with the *S. chilense* introgressions for *Ty*-*1*/*Ty*-*3* and *Ty*-*4*, which hampered cultivar development for many years (Hutton et al. 2015; Verlaan et al. 2011). Interestingly, there is no apparent linkage drag associated with *Ty*-*6*, and recent surveys of UF/IFAS begomovirus-resistant breeding lines indicate that this allele was maintained through many cycles of horticultural selection (S. Hutton, unpublished data). These data suggest that if *S. chilense* is in fact the source of *Ty*-*6*, the introgression may be very small, and it may be contained within a region that is highly syntenic to cultivated tomato and suffers no homeologous suppression of recombination. Another possibility is that the *Ty*-*6* resistance is derived from sources other than *S. chilense* such as cv. ‘Tyking.’

Considering the broad range efficacy *Ty*-*6* confers against mono- and bipartite begomoviruses, as well as the complementary resistance it provides in combination with other genes, *Ty*-*6* will likely prove extremely useful for many tomato breeding programs throughout the world. Although some of the markers used in this study were associated with *Ty*-*6*, none of those can be broadly applied in marker-assisted breeding, for reasons mentioned earlier. Further research is currently underway to generate whole genome resequencing data for several *Ty*-*6* inbred lines. These results should lead to the discovery of additional sequence polymorphisms than can be used for developing improved markers for use in breeding, in identifying the origin of the gene and in fine mapping the locus.

### Author contribution statement

JWS, SFH, HS and RS contributed to conception of experiments and acquired phenotypic data. EO, CS, DMF and S-CS designed genotyping assays and produced genotypic data. UG and SFH were responsible for data analysis.

## Electronic supplementary material

Below is the link to the electronic supplementary material.

Supplementary Fig. S1 Genetic map displaying centimorgan (cM) positions of SNP markers genotyped on F2 population from the cross between Fla. 8383 and Fla. 7776 (A). LOD plot of tomato chromosomes generated by QTL cartographer indicating a single QTL on chromosome 10 (B). (TIFF 4923 kb)

Supplementary material 2 (XLSX 42 kb)

Supplementary material 3 (DOCX 26 kb)
