# Supplementary figures and images for: Ty-6, a major begomovirus resistance gene on chromosome 10, is effective against Tomato yellow leaf curl virus and Tomato mottle virus
Source: Theor Appl Genet. 2019 Feb 13;132(5):1543–54. doi: 10.1007/s00122-019-03298-0 (PMC6476845; doi:10.1007/s00122-019-03298-0)

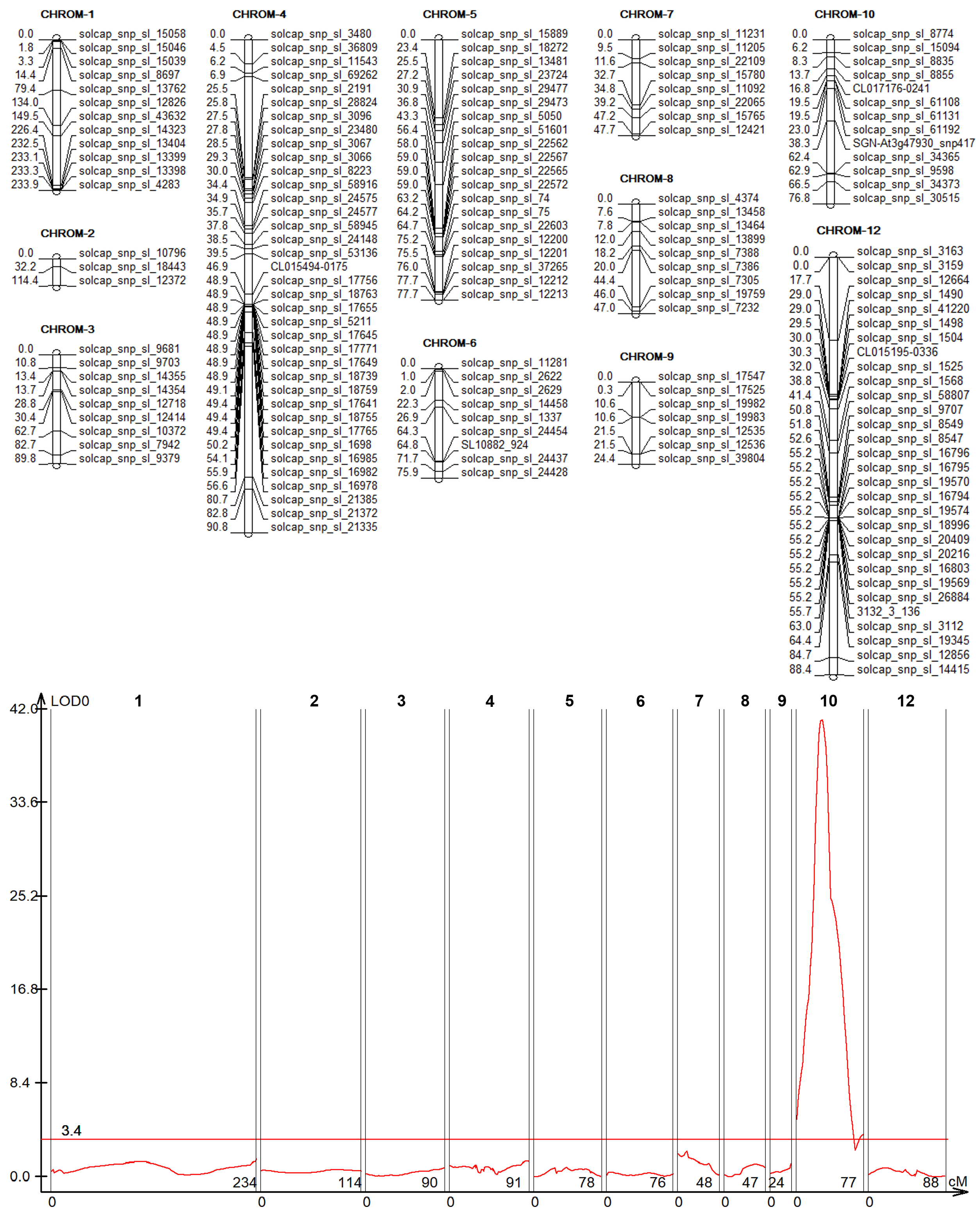

Supplement: Supplementary file 1 — Genetic map displaying centimorgan (cM) positions of SNP markers genotyped on F2 population from the cross between Fla. 8383 and Fla. 7776 (A). LOD plot of tomato chromosomes generated by QTL cartographer indicating a single QTL on chromosome 10 (B). (TIFF 4923 kb) [file 122_2019_3298_MOESM1_ESM.tif]
