# Supplementary material for: Ty-6, a major begomovirus resistance gene on chromosome 10, is effective against Tomato yellow leaf curl virus and Tomato mottle virus
Source: Theor Appl Genet. 2019 Feb 13;132(5):1543–54. doi: 10.1007/s00122-019-03298-0 (PMC6476845; doi:10.1007/s00122-019-03298-0)
Supplement: Supplementary file 3 — Supplementary material 3 (DOCX 26 kb) [file 122_2019_3298_MOESM3_ESM.docx]

**Title:** *Ty-6*, a major begomovirus resistance gene on chromosome 10, is effective against Tomato Yellow Leaf Curl Virus and Tomato Mottle Virus

**Journal:** Theoretical and Applied Genetics

**Authors:** Upinder Gill^1^, Reza Shekasteband^1^, Jay W. Scott^1^, Eben Ogundiwin^2^, Cees Schuit^3^, David M. Francis^4^, Sung-Chur Sim^4,5^, Hugh Smith^1^, Samuel F. Hutton^1^

**Affiliations:**

^1^Gulf Coast Research and Education Center, Institute of Food and Agricultural Sciences, University of Florida, 14625 CR 672, Wimauma, FL 33598-6101

^2^Nunhems USA, Inc., 890 Embarcadero Drive, West Sacramento, CA 95605

^3^Bejo Zaden, 1749 ZH Warmenhuizen, The Netherlands

^4^Department of Horticulture and Crop Science, The Ohio State University, Ohio Agricultural Research and Development Center, 1680 Madison Ave. Wooster, OH 44691, USA

^5^Department of Bioresources Engineering, Sejong University, 209 Neungdong-ro, Gwangjin-gu, Seoul, 05006, Korea

**Corresponding author:** Samuel F. Hutton; sfhutton@ufl.edu; Phone: 813-419-6610

**Supplementary Table S1:** Genetic background and *Begomovirus* resistance information of tomato breeding lines used in this study.

| **Breeding line** | **Genetic background^a^** | ***Begomovirus* resistance genes** | **TYLCV^b^** | **ToMoV^b^** | **Reference^c^** |
| --- | --- | --- | --- | --- | --- |
| Fla. 8680 | *S. chilense* LA2779; *S. lycopersicum* (UF/IFAS, Heb. Natl. Univ.) | *Ty-3, Ty-6* | R | R | Hutton et al. 2015 |
| Fla. 8383 | *S. chilense* LA2779; *S. lycopersicum* (UF/IFAS, Heb. Natl. Univ.) | *Ty-6* | MR | R | n/a |
| Fla. 8503C | *S. chilense* LA2779; *S. lycopersicum* (UF/IFAS and Heb. Natl. Univ.) | *Ty-6* | MR | R | n/a |
| Fla. 8472 | *S. chilense* LA1938; *S. lycopersicum* (‘Tyking’; UF/IFAS, NCSU) | *ty-5, Ty-6* | R | R | Scott et al. 2015 |
| Fla. 8638B | *S. chilense* LA1938; *S. lycopersicum* (‘Tyking’; UF/IFAS) | *ty-5, Ty-6* | R | R | Scott et al. 2015 |
| Fla. 8382B | *S. chilense* LA1938; *S. lycopersicum* (‘Tyking’; UF/IFAS, NCSU) | *ty-5, Ty-6* | R | R | n/a |
| Fla. 7776 | *S. lycopersicum* (UF/IFAS) | -- | S | S | Scott et al. 2006 |
| Fla. 7060 | *S. lycopersicum* (UF/IFAS) | -- | S | S | n/a |
| Fla. 7987 | *S. lycopersicum* (UF/IFAS) | -- | S | S | n/a |
| Fla. 8059 | *S. lycopersicum* (UF/IFAS) | -- | S | S | Scott et al. 2008 |
| Fla. 8044 | *S. lycopersicum* (UF/IFAS) | -- | S | S | n/a |
| Fla. 7781 | *S. lycopersicum* (UF/IFAS) | -- | S | S | Scott et al. 2000 |

^a^ UF/IFAS = University of Florida, Institute of Food and Agricultural Sciences, FL, USA; Heb. Natl. Univ. = Hebrew National University, Israel; NCSU = North Carolina State University, NC, USA; ‘Tyking’ = Hybrid cultivar from Royal Sluis, The Netherlands.

^b^ R = Resistant; MR = Moderately resistant; S = Susceptible.

^c^ References cited in Supplementary Table 1 are listed below:

Hutton SF, Ji Y, Scott JW (2015) Fla. 8923: A tomato breeding line with *Begomovirus* Resistance Gene *Ty-3* in a 70-kb *Solanum chilense* Introgression. HortScience 50 (8):1257-1259

Scott JW, Baldwin EA, Klee HJ, Brecht JK, Olson SM, Bartz JA, Sims CA (2008) Fla. 8153 hybrid tomato; Fla. 8059 and Fla. 7907 breeding lines. HortScience 43(7):2228-2230

Scott JW, Hutton SF, Freeman JH (2015) Fla. 8638B and Fla. 8624 tomato breeding lines with begomovirus resistance genes *ty-5* plus *Ty-6* and *Ty-6*, respectively. HortScience 50 (9):1405-1407

Scott JW, Jones JP (2000) Fla. 7775 and Fla. 7781: Tomato breeding lines resistant to fusarium crown and root rot. HortScience 35(6):1183-1184

Scott JW, Olson SM, Bryan HH, Bartz JA, Maynard DN, Stoffella PJ (2006) ‘Solar Fire’ Hybrid Tomato: Fla. 7776 Tomato Breeding Line. HortScience 41(6):1504-1505

**Supplementary Table S3:** Description of proprietary molecular markers provided by Bejo Seeds, Inc. and Nunhems USA, Inc. and used for genetic mapping of *Ty-6*.

| **Marker Name** | **Physical location (SL3.0)**  **(Megabase)** | **Marker source** | **Similarity among markers** |
| --- | --- | --- | --- |
| B_01 | 8.2 | Bejo Seeds, Inc. | N_06 |
| B_02 | 63.3 | Bejo Seeds, Inc. | N_18 |
| B_03 | 63.5 | Bejo Seeds, Inc. |  |
| B_04 | 63.6 | Bejo Seeds, Inc. | N_20 |
| B_05 | 63.9 | Bejo Seeds, Inc. | N_25 |
| B_06 | 64.0 | Bejo Seeds, Inc. |  |
| B_07 | 64.1 | Bejo Seeds, Inc. | N_28 |
| B_08 | 64.3 | Bejo Seeds, Inc. |  |
| B_09 | 64.5 | Bejo Seeds, Inc. |  |
| B_10 | 65.6 | Bejo Seeds, Inc. |  |
| B_11 | 65.6 | Bejo Seeds, Inc. |  |
| B_12 | 65.6 | Bejo Seeds, Inc. |  |
| B_13 | 65.6 | Bejo Seeds, Inc. |  |
| B_14 | 63.7 | Bejo Seeds, Inc. |  |
| B_15 | 63.7 | Bejo Seeds, Inc. |  |
| B_16 | 65.1 | Bejo Seeds, Inc. |  |
| N_01 | 4.8 | Nunhems USA, Inc. |  |
| N_02 | 5.1 | Nunhems USA, Inc. |  |
| N_03 | 5.1 | Nunhems USA, Inc. |  |
| N_04 | 5.5 | Nunhems USA, Inc. |  |
| N_05 | 7.5 | Nunhems USA, Inc. |  |
| N_06 | 8.2 | Nunhems USA, Inc. | B_01 |
| N_07 | 28.4 | Nunhems USA, Inc. |  |
| N_08 | 47.6 | Nunhems USA, Inc. |  |
| N_09 | 47.8 | Nunhems USA, Inc. |  |
| N_10 | 50.7 | Nunhems USA, Inc. |  |
| N_11 | 50.7 | Nunhems USA, Inc. |  |
| N_12 | 50.7 | Nunhems USA, Inc. |  |
| N_13 | 50.8 | Nunhems USA, Inc. |  |
| N_14 | 50.8 | Nunhems USA, Inc. |  |
| N_15 | 52.0 | Nunhems USA, Inc. |  |
| N_16 | 53.5 | Nunhems USA, Inc. |  |
| N_17 | 53.5 | Nunhems USA, Inc. |  |
| N_18 | 63.3 | Nunhems USA, Inc. | B_02 |
| N_19 | 63.5 | Nunhems USA, Inc. |  |
| N_20 | 63.6 | Nunhems USA, Inc. | B_04 |
| N_21 | 63.6 | Nunhems USA, Inc. |  |
| N_22 | 63.6 | Nunhems USA, Inc. |  |
| N_23 | 63.8 | Nunhems USA, Inc. |  |
| N_24 | 63.9 | Nunhems USA, Inc. |  |
| N_25 | 63.9 | Nunhems USA, Inc. | B_05 |
| N_26 | 63.9 | Nunhems USA, Inc. |  |
| N_27 | 64.1 | Nunhems USA, Inc. |  |
| N_28 | 64.1 | Nunhems USA, Inc. | B_07 |
| N_29 | 64.2 | Nunhems USA, Inc. |  |
| N_30 | 64.5 | Nunhems USA, Inc. |  |
| N_31 | 64.5 | Nunhems USA, Inc. |  |
| N_32 | 64.7 | Nunhems USA, Inc. |  |
| N_33 | 64.7 | Nunhems USA, Inc. |  |
| N_34 | 65.4 | Nunhems USA, Inc. |  |
| N_35 | 65.6 | Nunhems USA, Inc. |  |

**Supplementary Table S4:** Molecular markers used for genotyping *Ty-3*, *ty-5*, and *Ty-6* genes in breeding populations.

| **Marker name** | **Gene detected** | **Forward primer**  **(5´→3´)** | **Reverse primer**  **(5´→3´)** | **Enzyme** | **Marker type^a^** |
| --- | --- | --- | --- | --- | --- |
| TY3-5 | *Ty-3* | TGGGTGATCCGTTGATTGAAG | TGCCAGTACACGGTGGTTTT | - | SCAR |
| TY5.2 | *ty-5* | GCTTCGCGTTTGATCACAGT | AGGCGGTACTCGTGCATAAT | TaqI | CAPS |
| *Sl*NAC1 | *ty-5* | TGCCTGGTTTCTGCTGTCA | TAAAGCTGAAGAAGGACTTACCCT | TaqI | CAPS |
| UF_10.61192 | *Ty-6* | CATAAAGTTCCGGCGAGTGT | TCCATTCCAAACCAAGTGAAG | BssHII | CAPS |

^a^ SCAR= Sequence characterized amplified region; CAPS= Cleaved amplified polymorphic sequence marker.
